# Deep Learning-Based Defect Prediction for Mobile Applications

**DOI:** 10.3390/s22134734

**Published:** 2022-06-23

**Authors:** Manzura Jorayeva, Akhan Akbulut, Cagatay Catal, Alok Mishra

**Affiliations:** 1 Department of Computer Engineering, Istanbul Kültür University, Istanbul 34158, Turkey; 1800004575@stu.iku.edu.tr (M.J.); a.akbulut@iku.edu.tr (A.A.); 2 Yazara Payment Solutions Inc., 230 Park Avenue, 4th Floor, New York, NY 10169, USA; 3 Department of Computer Science and Engineering, Qatar University, Doha 2713, Qatar; ccatal@qu.edu.qa; 4 Faculty of Logistics, Molde University College—Specialized University in Logistics, 6410 Molde, Norway; 5 Department of Software Engineering, Atilim University, Ankara 06830, Turkey

**Keywords:** software defect prediction, software fault prediction, mobile application, Android applications, deep learning, machine learning

## Abstract

Smartphones have enabled the widespread use of mobile applications. However, there are unrecognized defects of mobile applications that can affect businesses due to a negative user experience. To avoid this, the defects of applications should be detected and removed before release. This study aims to develop a defect prediction model for mobile applications. We performed cross-project and within-project experiments and also used deep learning algorithms, such as convolutional neural networks (CNN) and long short term memory (LSTM) to develop a defect prediction model for Android-based applications. Based on our within-project experimental results, the CNN-based model provides the best performance for mobile application defect prediction with a 0.933 average area under ROC curve (AUC) value. For cross-project mobile application defect prediction, there is still room for improvement when deep learning algorithms are preferred.

## 1. Introduction

Mobile applications are rapidly evolving. Most previous applications were developed for the management of emails, contacts, calculations, and schedules, but nowadays, we see very diverse mobile applications that change our lives dramatically. Technology has also rapidly changed during the last decade, which eventually affected the mobile application domain.

Technologies such as cloud computing, Internet of Things (IoT), and Blockchain have helped mobile application developers to develop productivity applications that we did not anticipate in the past. For instance, with the help of cryptocurrencies and/or Blockchain infrastructure, people can easily transfer their savings (e.g., USD) from one country to another because different banks have already started to set up Blockchain networks (e.g., Ripplenet) that allow international money transfer between banks and no longer need specific protocols, which affect the transfer duration. Nowadays, international money transfers are very fast, like many other activities that we perform these days.

Different mobile applications have been developed recently. For instance, people started to discover that smartphones have more functional features, such as the use of a mobile sales assistant with near-field communication (NFC), which enables mobile devices to identify products by code and display them on mobile devices. This application has enabled people to reach the product they want faster [1]. The other benefit of mobile applications is ability to process the credit card information for payment transactions or to use them for faster payments. The last one is a SoftPOS solution that converts NFC-enabled Android devices to the normal POS terminals without the need for any additional device. This enables customers to experience in-store shopping while shopping from anywhere. Since Android devices can be bought by anyone, small businesses can benefit from this solution at low costs.

In parallel to this rapid progress in software development technologies, user expectations are also increasing. A small bug in an application affects the reputation of the software company detrimentally, users have no tolerance for the faults and failures. When a mobile application software is defective, it affects user experience and software development companies’ reputation dramatically [2]. Insufficient testing in mobile applications can cause serious problems after release [3]. A defective application cause different difficulties and waste time [4]. To minimize the number of faults and failures, analyzing previous mobile application defects can provide the possibility of improving them before deployment [5].

So far, software fault analysis and prevention have been performed using different techniques, such as static code inspection, early prototyping, adapting fault-tolerant design, using defensive programming, glass-box and black-box testing, and test automation. Alternatively, predictive models use past software metrics and data to predict faulty components for the next software releases. When there is previous fault data in previous versions, these defect data are used together with software metrics. Most models so far use machine learning algorithms. While there have been many different models developed for desktop and web applications, the number of models for mobile applications is rather limited and performance is not very satisfactory.

This study aims to develop a fault prediction model for mobile applications. We focused on Android mobile applications because they are open source and experiments are repeatable. In this research, we developed our models using deep learning and shallow learning techniques and applied them to 14 Android application datasets to show the performance of the models. We designed different deep learning-based models for software fault prediction in mobile applications. We evaluated the following research questions in this research:

- RQ1. To what extent can we predict the software faults using cross-project for mobile apps using deep learning approaches? Here, the goal is to understand how deep learning algorithms perform regarding mobile applications.
- RQ2. Can we improve the performance of deep learning models using data balancing approaches? The aim of this research question is to evaluate the effect of data balancing techniques.
- RQ3. Can we improve the performance of deep learning-based fault prediction models using feature engineering techniques?
- RQ4. To what extent can LSTM-based deep learning architecture predict software faults for mobile apps?

The paper is categorized as follows: The Section 2 presents the background and related work. Section 3 discusses the research methodology. Section 4 shows the model development. Section 5 explains the experimental results. Section 6 shows the discussion and Section 7 presents the conclusions.

## 2. Background and Related Work

A study on the identification of defects in software was presented by Akiyama [6]. However, the initial defect prediction studies using machine learning techniques started in early 2000s. Our methodology is different than the study of Akiyama because neither machine learning nor deep learning algorithms were applied by Akiyama [6] at that time. Recently, the mobile phone market has undergone a huge evolution. Very diverse mobile applications have been developed. However, there are still unrecognized defects of mobile applications that can affect businesses. To avoid this, defects of applications should be reviewed before releases. The benefit of these prediction models is that more testing resources can be allocated to fault-prone modules effectively.

Software engineers first test the functionality of code on the local systems to compare actual and expected results. When they find the difference between actual and expected code it becomes a defect. Software defects affect software quality. When a test engineer is testing code and finds the difference between actual and expected results, this is a bug. Error is a mistake in the code; while writing code, developers are not able to run or compile code. Failure is when the program is ready and customer-facing issues are in production. There are a few types of software defects: errors of commissions, errors of omission, errors of clarity, errors of speed, and capacity. The error of commissions means the error in command. The error of omission means the closing of a bracket was forgotten, left, or excluded. The error of clarity means a misunderstanding between developer and customer. The error of speed or capacity means the program works but not in the allowed time.

Machine learning uses various algorithms to analyze and learn data then make predictions and take decisions for future. Nowadays machine learning is a well-known technique and used in different areas, such as healthcare [7,8,9,10], industrial applications [11], banking [12], telecommunication [13,14], software development [15], etc. Machine learning is also used for regression tasks and defective classes (i.e., binary class classification). Machine learning has the following classifications: in supervised learning, it practices labelling data to detect traits. Unsupervised learning evaluates non-labeled data to identify hidden structures through determining the correlations of features. The algorithm can benefit from a limited number of labeled text documents for clustering and dimensionality reduction. Semi-supervised learning is used when data points are unlabeled, and only a tiny percentage of data is labeled. The last category is reinforcement learning, which helps to solve problems with trial and error.

Even though software architecture has shifted from monolithic applications to microservice architecture [16] today, software fault prediction is always a problem to be solved. All related articles published in the last three decades on software defect prediction and mobile applications defect prediction research have been extensively studied. Nevertheless, only exceptionally were articles published after the first decade of our century. Alsolai et al. analyzed publications focused on the defect prediction of OO Software systems using applied machine learning techniques and proposed a systematic literature review. The authors used private datasets and affairs on publicly available datasets. They applied to model k-fold cross-validation methods and detailed regression tasks. Additionally, various studies implemented unique models [17]. Perez and Tah developed a mobile application to detect defects on buildings using deep learning techniques. The application worked with a smartphone camera to recognize various problems on buildings [18]. Zhao et al. (2022) proposed a model for imbalanced datasets of 15 Android applications. They used feature learning with loss function into deep learning for imbalance issue and their model performance was better when compared to 25 defect prediction models [19]. Dong et al. (2017) proposed a study on the defect prediction of Android apk files. They used deep neural network for 50 Android apk files and obtained 85.98% accuracy. Deep neural networks achieved better results than traditional machine learning techniques [20]. Cheng et al. (2022) performed a model for a cross-project to predict defects of code committed before new releases of Android applications. They used adversarial learning and experimented on 14 Android applications. This model gives better results when compared with 20 others [21].

Kaya et al. presented a study and defect prediction model on machine learning and software metrics using data sampling methods. They described the point of data sampling methods for imbalanced datasets to improve defect prediction performance. They mentioned the Adaboost algorithm as the best for defect prediction [22]. Kaur et al. performed a study for mobile app defect prediction, which used publicly available datasets. Process metrics-based machine learning models were named as the best predictors [23]. Zhao et al. created a model for Android application defect prediction. To 12 applications datasets they applied loss function for imbalance class problems and deep learning models for defect prediction. They pointed out that IDL model performance is better than other models [24]. Sewak et al. researched different variations of LSTM and presented a study on malware detection using LSTM networks [25]. Most of these models used traditional machine learning algorithms. Only a few studies used deep learning algorithms for model development. In our model, we use artificial neural networks, convolutional neural networks, and long short term memory to solve defect prediction for Android applications.

## 3. Research Methodology

**Figure 1:**
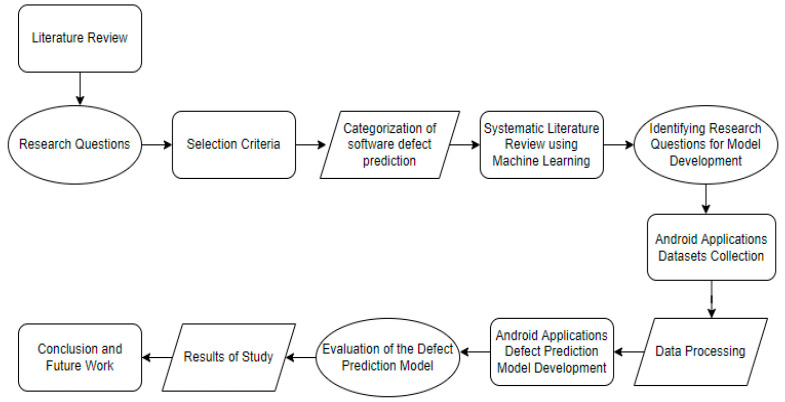
Methodology of SLR.

In order to make design decisions for the model to be developed in this study, we conducted a systematic literature review (SLR) study on this topic [26]. We identified nine research questions and applicable papers were reclaimed from digital platforms. We applied selection criteria to the selected studies and organized them for quality assessment. Each article was carefully considered according to quality assessment questions. First, we reviewed previous studies on software defect prediction and categorized them into three groups. Web applications, mobile Android applications, and Windows phone application defect prediction. There were many studies on web application defect prediction; however, for Windows phone application there were only limited resources, and for Android mobile application defect prediction only a few studies had been published. We carefully studied each publication and performed a study on mobile application defect prediction using machine learning methods. From obtained results in the literature review, we decided to develop a model for Android application defect prediction using deep learning techniques because studies on this are very limited. For the model development process, we first identified three research questions. We reviewed previous studies to identify models developed for Android application predictions. We recognized that only a few deep learning algorithms were applied. Therefore, we collected Android application datasets, completed data processing, and developed a model using deep learning algorithms. We attempted to answer the identified research questions after the model development process was completed. Figure 1 shows the methodology of this study.

## 4. Model Development

The goal of this study was to develop a model for mobile application defect prediction using deep learning algorithms. We used Python data science libraries, NumPy for numerical processing, pandas for data analysis, Matplotlib for visualization, and sklearn for processing machine learning datasets. Machine learning is the practice of using algorithms to analyze data, learn from that data, and then decide on or predict new data. In machine learning, we can use different algorithms to classify and predict the desired output. However, machine learning algorithms do not consider the context of the word as a sequence. It works based on the number of times the word has been repeated, a probability is derived, and then it performs a classification task. Deep learning learns a pattern of a word or a sequence and then tries to predict the desired outcome of the task. For deep learning, we prefer Keras API Classes. To use Estimator API, we define a list of feature columns, create the Estimator model, create a data input function, call, train, evaluate, and predict methods on the Estimator object.

### 4.1. Datasets

We used 14 available open-source Android application datasets from previous studies [27]. The datasets are available on the COMMIT GURU platform [28]. The datasets are numerical, of different sizes, and include six columns and 34.042 lines. Table 1 shows the names of datasets, web addresses, and the total number of downloads.

### 4.2. Data Processing

**Figure 2:**
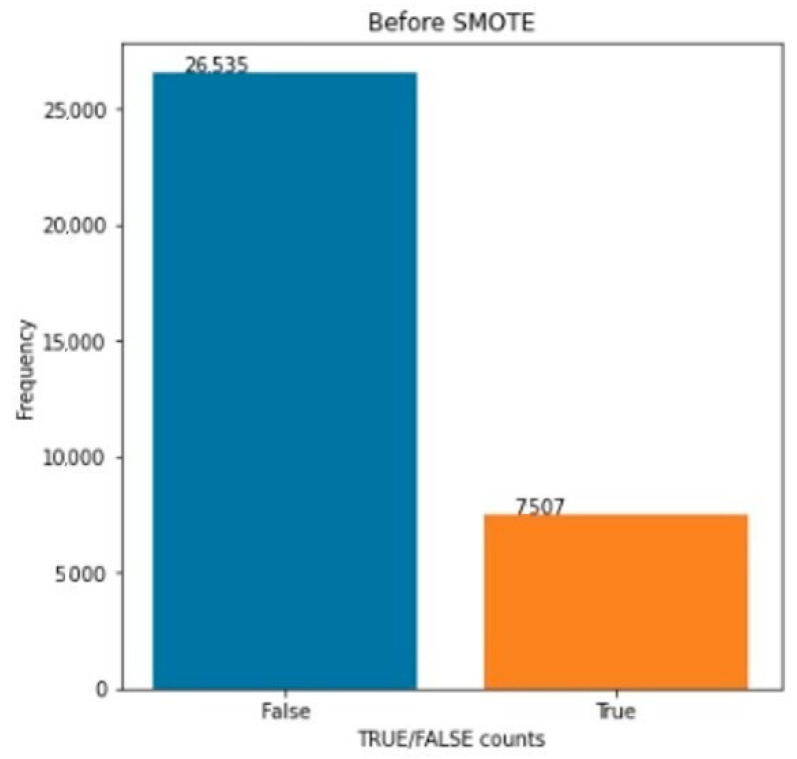
Dataset classes distribution.

We controlled the number of examples belonging to the true or false class to identify dataset types. The dataset is imbalanced (false class is larger than true class), as shown in Figure 2. Then we searched for null values in the datasets, as if datasets contain nonvalues, it will create a problem. We changed null values belonging to the feature columns, which we filled with zero values (zero means this feature has no effects on prediction) and false for the Contains Bug folder. Then we split the dataset X-feature columns, Y-class columns, data X-input, and data Y-output.

**Figure 3:**
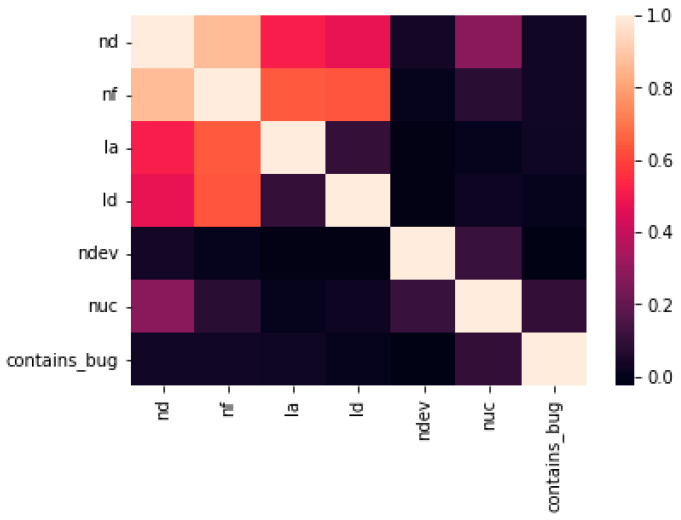
Correlation between features.

We used a labeling approach to encode specific numbers (0,1,2) and sklearn preprocessing technique to transform 0 as false and 1 for true. After this, we identified our datasets into train and test in each loop: X_train, X_test, Y_train, Y_state, X features, and Y classes. We used a random state as the parameter of test split, each time the cells were run if we had 1000 examples in the dataset, the random state would randomly choose 80 percent of the training and test datasets. We checked X_train and shaped X_test. In order to see how many shape examples we obtained: we split and obtained the size. The correlation between every feature is shown in Figure 3.

### 4.3. Validation

We applied a 10-fold cross-validation to the dataset to evaluate the performance of our model. The accuracy of the random state might not show the actual performance. Yet applying cross-validation, we can resolve this problem. We split the dataset into 10 folds, and cross-validation uses 10 blocks to train the methods and then the last block to test the method and keep track how of how well the method did with the test data. From the training set, we obtained the best learning result from the test’s best validation result. We believe that the assessment of our model will be more accurate when using a 10-fold cross-validation.

### 4.4. Feature Engineering

We used supervised deep learning to predict features in the dataset. Features have two critical properties: units and the magnitude computed with them. When we have a different feature in the dataset, it is calculated by various units and magnitudes. To run data into a neural network, we had to scale it using two techniques—normalization and standardization used for data scaling. Normalization scales down data between 0 to 1 and standardization helps to scale down data based on a standard normal distribution. We needed to scale down values between 0 to 1 in deep learning. In convolutional neural network images, pixels are between 0 to 1. An artificial neural network, which uses TensorFlow and Keras libraries, except for input between 0 to 1, will help the learning of weights quickly. To achieve that, we used a MinMaxScaler. We created an instance of a MinMaxScaler and after this we fit our data. Fitting the data allows the model to know each column’s minimum and maximum values.

### 4.5. Data Balancing

**Figure 4:**
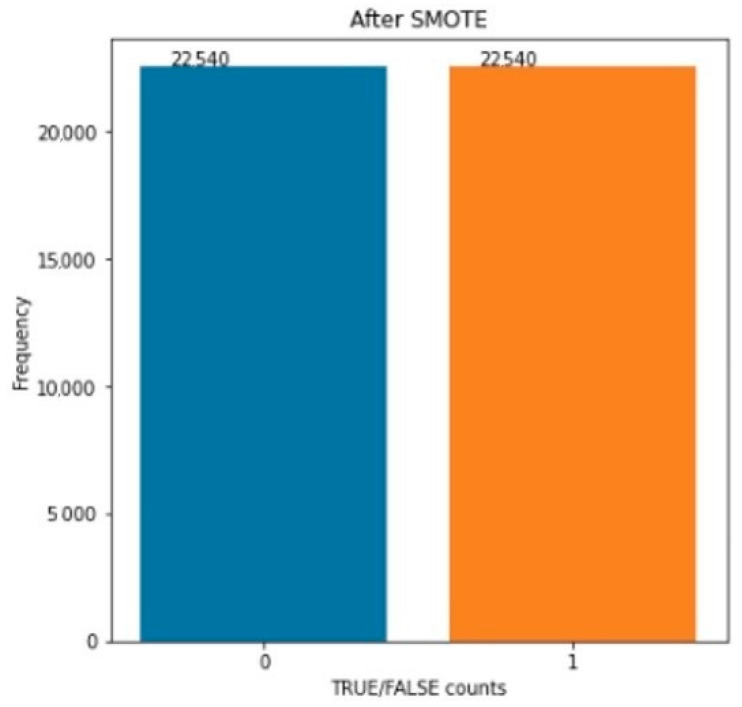
Data balancing after SMOTE.

As we detailed in previous sections, we are searching for solutions for the detection of mobile application defects. Additionally, we are working on Android applications’ numerical datasets. In binary classification, we ended up with one class, which is essential for this study. However, we do not have enough data. In our datasets, a majority class is more significant than a minority class, and we have a class imbalance problem. Our minority class is a focus class. There are a few techniques to create fake data and balance datasets: random oversampling—from the imblearn library—makes a new sample for the minority class by sampling with replacements. We discovered that in literature studies, RandomOverSampler reported that their model did not provide the best performance; therefore, we dropped this method. Random undersampling picks random samples of the majority class, and after sampling, the majority class and minority class have the same data points. The near-miss undersampling method uses KNN to perform undersampling. There are a few types of this method, near-miss selects positive samples from the nearest distance to the farthest distance and keeps negative samples and positive samples of the average of N closest and most significant. We focused on SMOTE because of its performance and used a Python imblearn library and the oversampling method SMOTE to balance the trained dataset and increase the minority class of the dataset in Figure 4. SMOTE takes each minority sample and introduces synthetic data points connecting the minority sample and its nearest neighbors. Neighbors of the K-nearest neighbors were chosen randomly, using the same random oversampling.

### 4.6. Deep Learning Algorithms

This section identifies algorithms used for the defect prediction model development of Android applications. We analyzed previous studies and identified most studies used traditional machine learning methods and focused on web and desktop applications. Deep learning algorithms were used in a few studies but for mobile applications, limited research was observed. We developed our model with deep learning algorithms and believe that the performance of our model can be used to solve the problems.

**Figure 5:**
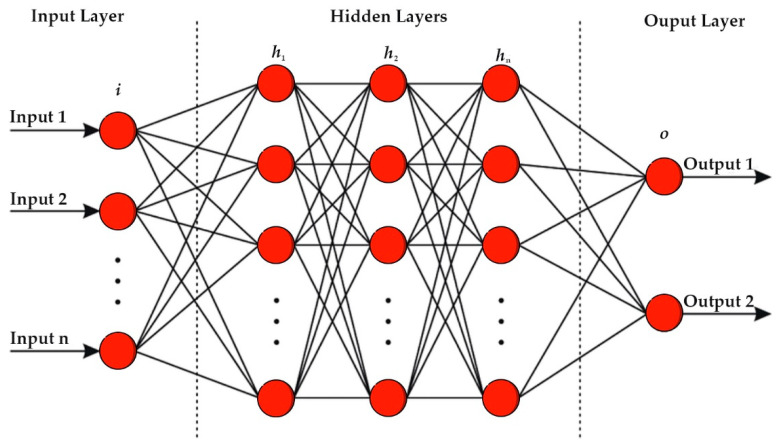
Artificial neural networks.

Artificial Neural Networks are computing systems inspired by the brain’s neural networks. These networks are based on a collection of connected units called artificial neurons. Each connection between neurons can transmit a signal from one neuron to another. The receiving neuron processes the signal and signals the downstream neurons connected to it. The structure of artificial neural networks has the following features: neurons organized in layers; input layer (absolute values from the data); hidden layers (layers between input and output, three or more hidden layers is “deep network”); and output layer (final estimate of the output). Artificial neural networks are typically organized in layers. Different types of layers include dense (or fully connected) layers, convolutional layers, pooling layers, and recurrent layers. Additional layers may perform various transformations on their inputs. The convolutional layer is most likely used with image data, the recurrent layer is used for time-series data, and the dense layer is a layer that connects each input to each output within its layer. We used the Keras library to build our neural network. Our model is sequential; we have an input layer, three hidden layers, and an output layer—our artificial neural network model is shown in Figure 5. We used a kernel initializer for weights and activation functions, such as Relu, SoftMax, and Sigmoid; we also used Adam optimizer and binary_crossentropy to evaluate our model. We started building our artificial neural network model with an input layer with six nodes because we have six columns in our dataset. Then we added hidden layers 12, 10, and 3 nodes and the output layers are one node. Our model trained for 100 epochs. An epoch is when all datasets pass a neural network, then continues. We needed to learn from the dataset and update the model. The batch size is how much data we want to process in a loop step by step. First ten samples are obtained, then the artificial neural network gives another ten until the end with randomly given samples. The architecture of our artificial neural network is shown in Figure 5. We performed tests on the dataset to predict accuracy. Following that, we evaluated the model with 10-fold cross-validation and calculated the ROC AUC, confusion matrix, and accuracy metrics.

**Figure 6:**
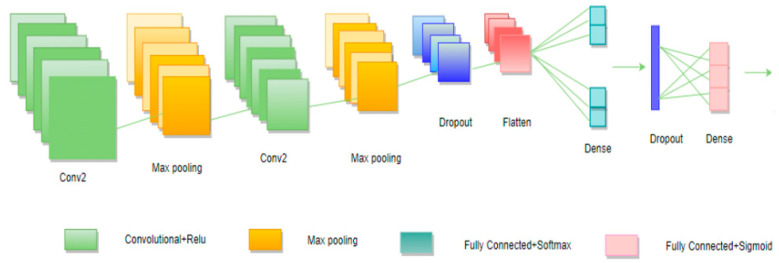
Convolutional neural networks.

CNN is an artificial neural network that is used for analyzing images. It is mostly used for image and vision-based data analysis or data classification problems. Most generally, we think of CNN as an artificial neural network specializing in picking out or detecting patterns or making sense of them. This pattern detection is what makes CNN useful for image analysis. Convolutional neural networks are just a form of artificial neural networks with different shapes from a standard MLP. CNN has hidden layers called convolutional layers, and it is precisely this layer that makes CNN. CNN has other non-convolutional layers, but the basis of a CNN is the convolutional layer. The convolutional layer receives input and then transforms and outputs the transformed input layer. With a convolutional layer, this transform is a convolutional operation. Recall that Tensors are N-dimensional arrays that we build up to: Scaler—3, Vector—[3–5], Matrix—([3,4], [5,6], [7,8]), Tensor—(([1,2], [3,4]), ([5,6], [7,8])). Tensors make it very convenient to feed in sets of images into our model—(I, H, W, C). I: images; H: height of image in pixels; W: width of image in pixels; C: color channels: 1-Grayscale, 3-RGB. To understand the difference between a densely connected neural network and a convolutional neural network, recall that we were already able to create a deep neural network with tf.estimator API. In a densely connected layer, every neuron in one layer is directly connected to every other neuron in another layer. For the convolutional layer, we take a different approach. Each unit is connected to a smaller number of nearby units in the next layer. Convolutions also have a significant advantage for image processing, where pixels nearby are much more correlated to each other for image detection. Each convolution neural network layer looks at an increasingly more significant image part. Having units only connected to nearby units also helps with regularization, limiting the search of weights to the size of the convolution. Convolutional neural networks did not exist before for mobile application defect prediction. We decided to build a sequential model using convolutional neural networks as well. We used Keras library, NumPy, sklearn libraries, Conv2D, MaxPooling2D, dropout, flatten, dense layers, and activation functions, such as Relu, SoftMax, and sigmoid. We reshaped our train and test data to make it easy to process our model efficiently. We compiled a model to measure performance with the Adam optimizer and used loss functions, such as binary cross-entropy. Convolutional neural network model parameters are presented in Figure 6. We started building our convolutional neural network with the sequential method and convolution layer. The convolution means core between blocks of CNN. We applied a 2D convolutional layer with 64 filters and one kernel size and we defined Relu (rectified linear unit activation function). Input data was multiplied by weights and the point of the training model was by adjusting weights and biases to find the appropriate values representing the training data. Neurons obtain a variety of signals and outputs the signals. The activation function decides whether to output the signals or not. There are a few types of activation functions. Sigmoid is used to predict the probability of output. Relu is used most in convolutional neural networks and other classifications. As the second layer, we applied the max pooling 2D layer with 1,6 filters in our model. The max pooling 2D operation takes the higher value from the convolutional layer and replaces it with the output. There are also different types of pooling layers, such as the mean pooling layer, which takes the low values from the convolutional layer output and replaces it with the mean pooling layer output. The third layer of our model is the convolutional 2D layer with 32 filters and one kernel size, and the activation function is Relu. Then we added the max pooling 2D layer with 1,1 filters. We used the dropout layer to reduce the overfitting problem. Then we applied the flatten layer to our model because the last layer is dense, and the artificial neural network requires values in a 1D feature vector. Flattening enables the output of the convolutional or max pooling layer to flatten all its structures in order to create a single feature vector that the dense layer can use for final classification. We added a dense layer with 250 nodes and the activation function SoftMax because it gives the best performance and then the dropout layer. The final output layer had one node and activation function Sigmoid. We compiled the model with the Adam optimizer based on datasets, and for deciding parameters, we used the loss function binary cross-entropy. We trained a convolutional neural network with 100 epochs and 10 batch sizes. Figure 6 shows the architecture of a convolutional neural network.

**Figure 7:**
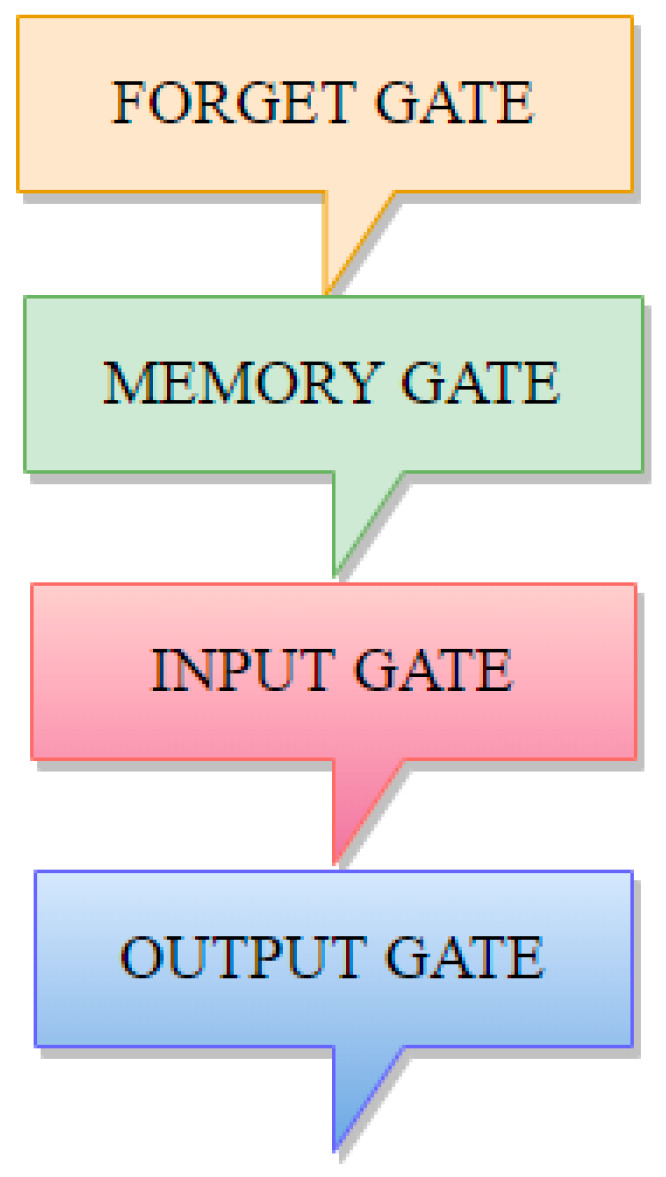
Long short term memory cells.

LSTM is an artificial recurrent neural network (RNN) architecture type, and it is used in deep learning. It allows neural networks to remember data and forget non-applicable data. We can say that long short term memory is a type of recurrent neural network. Recurrent neural networks are good for short context classification. In a recurrent neural network, processed input data after output is provided to the input in the next step and new input data. It allows a recurrent neural network to remember previous steps in the sequence. A typical LSTM network is shown in Figure 7.

**Figure 8:**
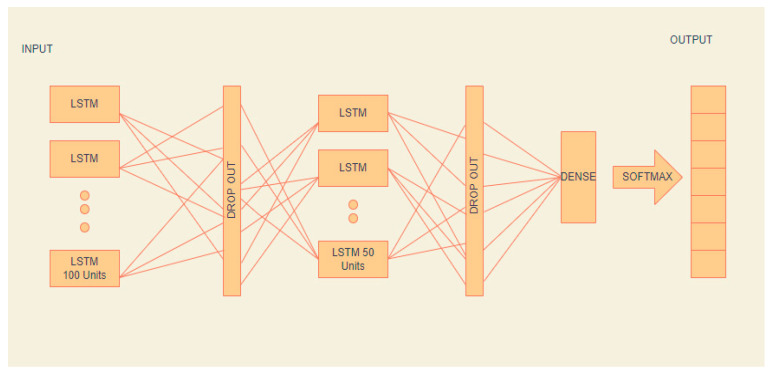
Long short term memory architecture.

However, in recurrent neural network, more data is a problem. It makes not effective in learning new data. Long short term memory is designed for those issues of recurrent neural networks. Long short term memory provides a solution for this problem; it is a named state. The state is a cell consisting of four parts and each part is a gate: forget gate, memory gate, input gate, and output gate. Forget gate is the sort of state in which data stored in the internal state can be forgotten and is no longer relevant. Input gate is what new data we should add or update into working storage data. Output gate is all the data stored in the state of which part should be output immediately. Numbers between 0–1 can be assigned to these gates; 0 means the gates are effectively closed, and one means the gate is wide open. We can make regularizations on them, add, or add a little bit. LSTM cells are shown in Figure 8.

We used the long short term memory model for Android application defect prediction. We imported Keras and TensorFlow libraries, and because LSTM expects data in a specific shape, we reshaped our data. We defined the num_of_samples, timesteps, nb_features, and nb_out. We started building our model with the sequential method and added the first LSTM layer with 100 units. We used true return sequences because we wanted each output signal to be returned to an input. The second layer used dropout to reduce overfitting. The third layer was LSTM, with 50 false units and returns sequences, then dropout layer was used.

**Figure 9:**
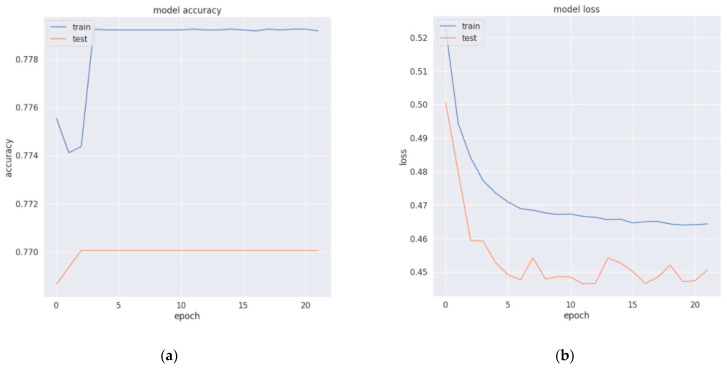
Long short term memory model (**a**) accuracy and (**b**) loss.

The last layer was dense with the activation function Sigmoid. We compiled our model with binary cross-entropy and the Adam optimizer and accuracy metrics. Figure 8 shows LSTM architecture. We trained our model and evaluated the results with 10-fold cross-validation. Model loss and accuracy curves are presented in Figure 9. Since there was a large gap between training and testing curves, we had to update the model settings for the last experiments. We were able to minimize this gap after the model parameter adjustments. Figure 9 belongs to the previous experimental settings, which were later updated during the fine-tuning of the model.

### 4.7. Accuracy Metrics

**Figure 10:**
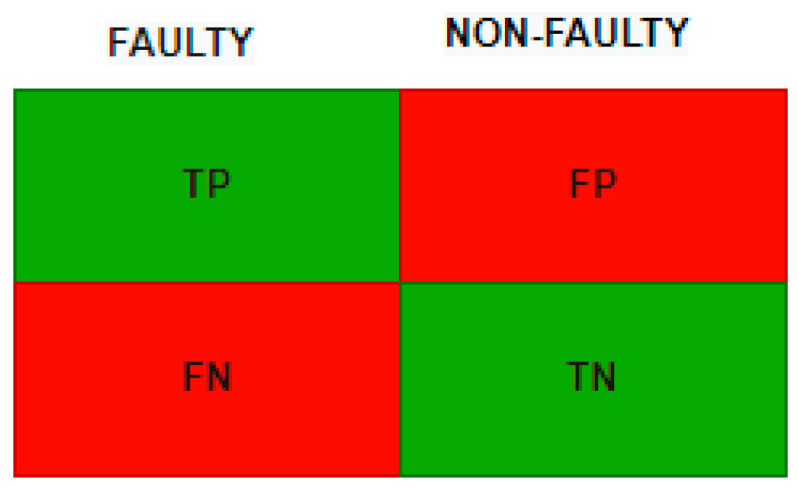
Confusion matrix example.

In this section, we explain accuracy metrics used in this study. We trained our model in training data and tested it with testing data. Now we need to summarize our predicted results. One way to achieve this is using the confusion matrix method. We have two categories, faulty and non-faulty, and in the last column we have true positives. True negatives (TN) refer to the non-faulty classes correctly identified by the algorithm; false negatives (FN) are faulty classes, but non-faulty classes predicted by the algorithm; false positives (FP) are non-faulty classes, but the algorithm predicts that they are faulty. Figure 10 shows the confusion matrix example.

**Figure 11:**
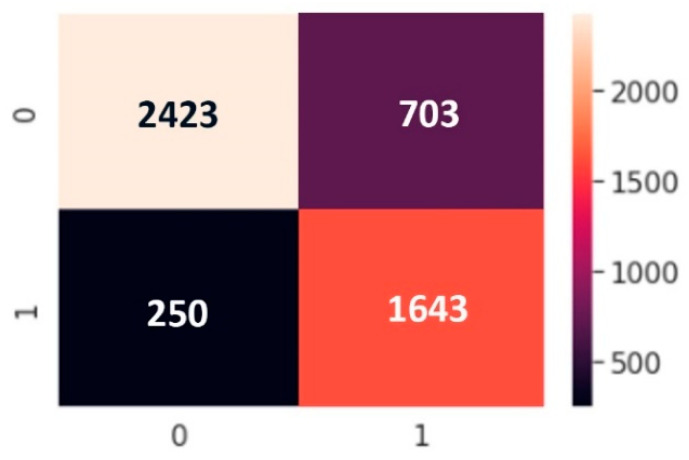
Confusion matrix of ANN model.

**Figure 12:**
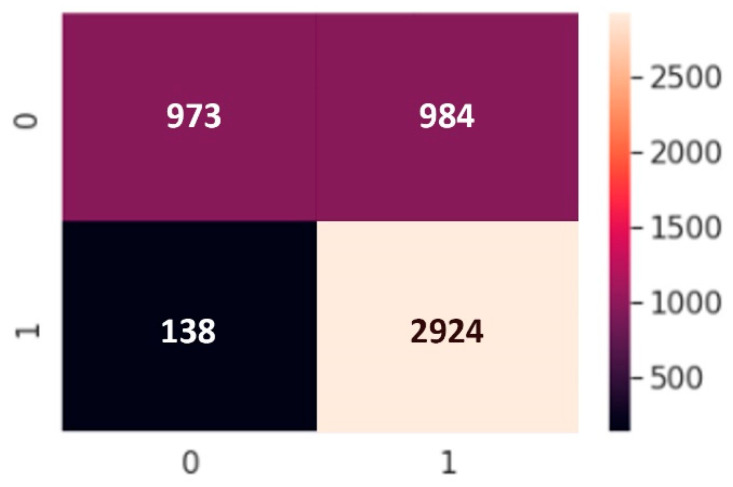
Confusion matrix of CNN model.

**Figure 13:**
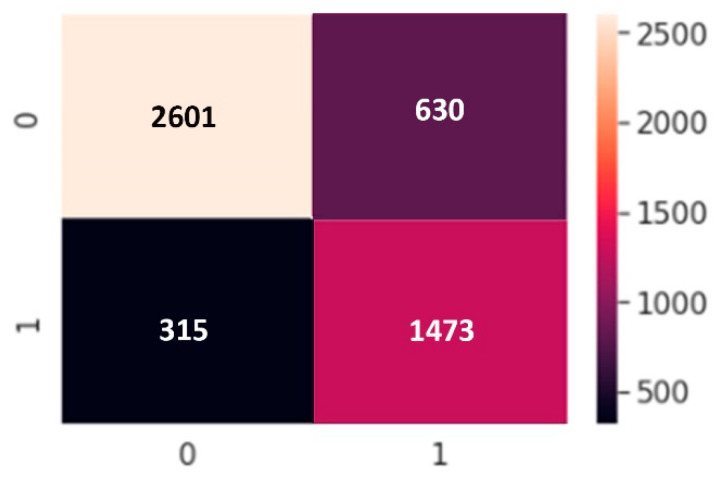
Confusion matrix of LSTM model.

We use a binary classification because of the dataset type. In classification, there are two statements: class labels and probability. We applied confusion matrix to see how many predicted classes were correctly predicted and how many were not. The *X*-axis is the prediction and the *Y*-axis is the class label. From a balanced dataset we can calculate the accuracy from the confusion matrix and obtain results but in an imbalanced dataset, values in categories are very different; one category can be maximum and another minimum. Our confusion matrix results are presented in Figure 11, Figure 12 and Figure 13.

Instead of our datasets, results show that true positives are more numerous than false positives. If we calculate accuracy with the Equation (1):

(1) $$\mathrm{Accuracy}=\frac{\mathrm{TP}+\mathrm{TN}}{\mathrm{TP}+\mathrm{FP}+\mathrm{FN}+\mathrm{TN}}$$

Accuracy alone is not enough to interpret the success of the model. We focused on recall and precision because these metrics are used for imbalanced datasets. In terms of recall, out of the total actual positive values, how many values did we correctly predict positively? Recall is also named the true positive rate or sensitivity. In terms of precision, out of the total predicted results, how many results are positive? Equation (2) of Precision and Recall is shown as follows:

(2) $$\mathrm{onRecall}=\frac{\mathrm{TP}}{\mathrm{TP}+\mathrm{FN}}\;\mathrm{Precision}=\frac{\mathrm{TP}}{\mathrm{TP}+\mathrm{FP}}$$

We applied the $F_{\beta }$ score because all values are important from the actual and predicted class. If we consider our beta value as 1, our $F_{\beta }$ becomes an F1-Score. The formula for $F_{\beta }$ is shown as follows:

(3) $$F_{\beta }=(1+\beta^{2})\;\frac{\mathrm{Precision}\times \mathrm{Recall}}{\beta^{2\;}\times \mathrm{Precision}+\mathrm{Recall}}$$

**Figure 14:**
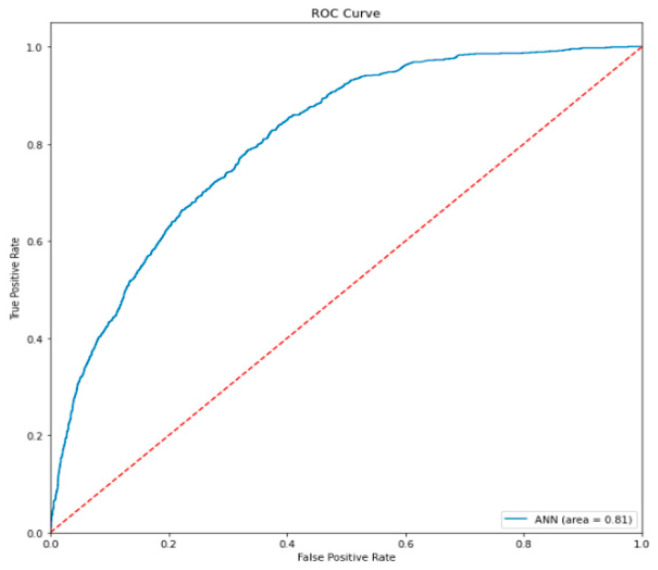
ROC and AUC curve.

We combined Precision and Recall in selecting the right metrics. A binary classification also uses ROC (receiver operating characteristics) and AUC (area under curve) performance metrics. If the area under the curve is higher, it means the model is better at predicting classes. It is mostly used in binary classification, such as fraud detection, spam detection, and software defect prediction. When AUC is 1, it means the model performs well, when model AUC is 0 or close to 0 then the model is performing at its worst separability. The ROC curve of the artificial neural network is shown in Figure 14.

## 5. Experimental Results

In this section, we summarize the obtained results for an Android application software defect prediction model. We used ANN, CNN, and LSTM algorithms in this research. We also applied cross-project analysis by combining all the datasets for training, except the test data, and for the test we chose a different dataset each time. We preferred deep learning algorithms because previous studies applied mostly machine learning classifiers. The experimental results of ANN are shown in Table 2, CNN results are presented in Table 3, LSTM results are shown in Table 4, and algorithm performance in datasets is shown in Table 5. We found these results by tuning the hyperparameters empirically. However, the use of metaheuristic algorithms could increase performance of the classifiers.

## 6. Discussion

In this section, we discuss our software defect prediction for Android application model performance. We evaluated three deep learning defect prediction models. We used 14 Android application projects from the COMMIT GURU platform. We used 10-fold cross-validation to evaluate the performance of each algorithm. We implemented SMOTE oversampling techniques to balance our datasets. We evaluated results with accuracy metrics, such as Recall, Precision, F1-score, ROC, and AUC. We evaluated the following research questions:

RQ1. To what extent can we predict the software faults using cross-project for mobile apps using deep learning approaches?

For this research question, we analyzed deep learning performance.

RQ2. Can we improve the performance of deep learning models using data balancing approaches?

For imbalanced mobile application datasets, we used SMOTE oversampling techniques to balance and improve the effectiveness of datasets.

RQ3. Can we improve the performance of deep learning-based fault prediction models using feature engineering techniques?

To improve the performance of deep learning models, we applied the data-scaling technique from input variables to an output variable. We used MinMaxScaler because our datasets are numerical, and we practiced binary classification. MinMaxScaler used for 0, 1 and for deep learning input variables expected in an 0, 1.

RQ4. To what extent can deep learning architecture predict software faults for mobile apps?

Specifically, we focused on CNN and LSTM deep learning algorithms because, based on recent review articles, they are the most preferred algorithms for software engineering problems, namely, malware detection [29] and phishing detection [30]. However, the other deep learning algorithms [31] might be also investigated regarding this problem and performance can be improved this way. Additionally, different handcrafted and non-handcrafted features can be analyzed [32]. We focused on Android applications in this research because the available datasets use Android projects and there were no publicly available datasets from other mobile operating systems. Additionally, we observed this issue in our recent review study on mobile malware detection [29], nearly all of the papers analyzed Android projects. However, the performance of the algorithms on different mobile operating systems, such as Windows, might be different. This requires additional research, which has not been performed in this study.

While SLR papers on mobile application defect prediction have been recently published [9], tertiary studies, such as [33], can be performed as well. Tertiary studies can synthesize secondary studies (i.e., SLR and systematic mapping (SM) studies) and present the state-of-the-art in this field. While finding relevant papers, we followed the SLR protocol carefully; however, due to some manual steps, some papers might have been neglected. A similar analysis using automated tools [34] could help to find more relevant papers in this research and include more deep learning algorithms. Previously, we performed different SLR studies [35,36] and observed the potential gaps in the relevant fields. In this research, we also used the knowledge of our recently published SLR paper [37]. Researchers can also examine this paper to see the potential research problems in this field.

## 7. Conclusions

Mobile applications have become a part of our lives. The goal of businesses is to propose users’ application with zero defects and make things easier. Mobile applications are tested manually and there might be unseen defects. Additionally, nowadays there are increasing types of mobile phones on the market, and mobile applications should be adapted for this. To recognize defects before the testing stage, the literature previously proposed defect prediction models for software using machine learning techniques. For mobile applications there were limited studies, and we proposed a study on software defect prediction models for mobile applications using deep learning methods. We used fourteen open-source Android application datasets of different sizes and platforms. We applied artificial neural networks, convolutional neural networks, and long short term memory. In an AUC-based performance benchmark, the CNN was the highest performing model with 0.93. We compared our results with open-source machine learning algorithms, such as support vector machines and decision trees, and our models have better results. In future work, we plan to use our defect prediction models in SoftPOS real-time applications, because the most important things are security and robust, stable application for SoftPOS.

## Figures and Tables

**Table 1:** Datasets.

Datasets	Repository	Lines	Downloads
Afwall	https://github.com/ukanth/afwall (accessed on 21 January 2022)	1025	500,000
Alfresco	https://github.com/Alfresco/alfresco-android-app (accessed on 21 January 2022)	1004	50,000
androidSync	https://figshare.com/s/9a075be3e1fb64f76b48 (accessed on 21 January 2022)	209	100,000
androidWallpaper	https://github.com/olivergeith/android_wallpaperDesigner (accessed on 21 January 2022)	588	5,000,000
anySoftKeyboard	https://github.com/AnySoftKeyboard/AnySoftKeyboard (accessed on 21 January 2022)	2971	25,271
Apg	https://github.com/thialfihar/apg (accessed on 21 January 2022)	3780	N/A
atmosphere	https://github.com/Atmosphere/atmosphere (accessed on 21 January 2022)	5474	1,000,000
chatSecure	https://github.com/guardianproject/ChatSecureAndroid (accessed on 21 January 2022)	2579	N/A
facebook	https://github.com/facebook/facebook-android-sdk (accessed on 21 January 2022)	548	5,000,000,000
flutter	https://github.com/flutter/flutter (accessed on 21 January 2022)	10,405	100,000
kiwis	https://github.com/kiwix/kiwix-android (accessed on 21 January 2022)	1373	1,000,000
owncloudandroid	https://github.com/owncloud/android (accessed on 21 January 2022)	3700	100,000
Pageturner	https://github.com/NightWhistler/PageTurner (accessed on 21 January 2022)	164	50,000
reddit	https://github.com/emmaguy/wear-notify-for-reddit (accessed on 21 January 2022)	222	50,000,000

**Table 2:** ANN defect prediction results (within-project analysis).

Datasets	Accuracy (%)	Precision	Recall	F-1 Score	AUC
aFall	65	0.73	0.69	0.80	0.91
Alfresco	68	0.74	0.70	0.77	0.92
androidSync	70	0.70	0.70	0.83	0.96
androidWalpaper	84	0.71	0.84	0.77	0.94
anySoftKeyboard	75	0.93	0.80	0.79	0.90
Apg	70	0.76	0.83	0.75	0.89
atmosphere	71	0.73	0.72	0.81	0.93
chatSecure	69	0.82	0.68	0.73	0.91
facebook	72	0.74	0.70	0.70	0.84
flutter	70	0.80	0.76	0.72	0.90
kiwis	67	0.71	0.90	0.69	0.93
owncloudandroid	70	0.72	0.80	0.71	0.96
Pageturner	68	0.73	0.76	0.73	0.92
reddit	72	0.70	0.71	0.77	0.90
Average	70.79	0.75	0.76	0.755	0.915

**Table 3:** CNN defect prediction results (within-project analysis).

Datasets	Accuracy	Precision	Recall	F-1 Score	AUC
aFall	67	0.73	0.70	0.69	0.95
Alfresco	70	0.67	0.80	0.71	0.96
androidSync	64	0.94	0.70	0.65	0.94
androidWalpaper	66	0.82	0.66	0.70	0.96
anySoftKeyboard	72	0.75	0.70	0.83	0.90
Apg	69	0.75	0.77	0.70	0.93
atmosphere	70	0.68	0.90	0.80	0.91
chatSecure	67	0.70	0.75	0.73	0.96
facebook	71	0.75	0.69	0.81	0.90
flutter	68	0.84	0.70	0.72	0.94
kiwis	73	0.76	0.74	0.69	0.92
owncloudandroid	70	0.80	0.72	0.69	0.96
Pageturner	69	0.75	0.82	0.70	0.90
reddit	70	0.73	0.69	0.85	0.93
Average	69	0.76	0.738	0.734	0.933

**Table 4:** LSTM defect prediction results (within-project analysis).

Datasets	Accuracy	Precision	Recall	F-1 Score	AUC
aFall	69	0.77	0.80	0.69	0.95
Alfresco	70	0.71	0.77	0.82	0.93
androidSync	73	0.83	0.79	0.65	0.86
androidWalpaper	68	0.80	0.93	0.77	0.94
anySoftKeyboard	71	0.73	0.69	0.80	0.91
Apg	72	0.74	0.80	0.77	0.90
atmosphere	69	0.70	0.72	0.71	0.89
chatSecure	73	0.80	0.75	0.72	0.95
facebook	70	0.72	0.74	0.83	0.90
flutter	67	0.70	0.76	0.74	0.92
kiwis	70	0.71	0.90	0.73	0.96
owncloudandroid	72	0.83	0.70	0.69	0.90
Pageturner	69	0.75	0.71	0.70	0.93
reddit	70	0.73	0.80	0.77	0.90
Average	70.21	0.751	0.775	0.742	0.917

**Table 5:** Cross-project analysis results (AUC).

Projects	ANN	CNN	LSTM
aFall	0.65	0.67	0.69
Alfresco	0.68	0.70	0.70
androidSync	0.70	0.64	0.73
androidWalpaper	0.84	0.66	0.68
anySoftKeyboard	0.75	0.72	0.71
Apg	0.70	0.69	0.72
atmosphere	0.71	0.70	0.69
chatSecure	0.69	0.67	0.73
facebook	0.72	0.71	0.70
flutter	0.70	0.68	0.67
kiwis	0.67	0.73	0.70
owncloudandroid	0.70	0.70	0.72
Pageturner	0.68	0.69	0.69
reddit	0.72	0.70	0.70
Average	0.71	0.69	0.70

## Data Availability

Not applicable.
